# The "Christian World" and Consumption Sanatoriums

**Published:** 1910-08-13

**Authors:** W. D. Wilkins

**Affiliations:** Benenden.


### Editor's Letter-Box.

### THE "CHRISTIAN WORLD" AND CONSUMPTION SANATORIUMS.

*To the Editor of* The Hospital.

Sir,—It is an amusing and significant fact that Mr. Alabone finds abuse of sanatorium treatment to be the most effective method of increasing his own *clientèle* and of establishing confidence in his own system of treatment.

It will, however, surprise no one who is at all conversant with the powers of logic possessed or rather not possessed by the type of patient that turns to the irregular practitioner for treatment, but it is certainly surprising to find that a journal of the standing of the *Christian World* should place confidence in the statements of a few dissatisfied patients, brought to their notice through the unbiased and disinterested medium of Mr. Alabone.

The value of sanatorium treatment is far from easy to decide by statistics, as there are so many pitfalls and fallacies to be avoided, and authorities, such as Dr. Karl Pearson, who have made a close study of the problem, have confessed their inability to come to a definite conclusion. How absurd it seems then that the treatment should be airily condemned because some patients are found who have not benefited by it, or have speedily relapsed. No advocate of the open-air treatment has ever claimed that all cases can be arrested or even improved, and failure in certain cases does not prejudice the case at all.

To anyone who has worked in a sanatorium proof of the efficacy of the treatment seems unnecessary, the results in suitable cases being so striking, and if unsuitable cases are not benefited we cannot be blamed for failing to perform what we do not claim to perform.

Relapse after return home is not a reason for condemning the treatment, for the fault lies with the social and political conditions which force a patient to return to the same unhealthy life and to take renewed draughts of the same poison that has already so severely affected him.

Undoubtedly there are discontented patients, and these we shall have always with us, for after the average patient has spent a few weeks at a sanatorium he feels strong and well, and begins to chafe at the necessary restrictions and the close adhesion to the hygienic rules which are really as essential to him as ever they were. But, on the other hand, it is, as you stated in your leader, a gross mis-statement to say that ex-patients are pretty unanimous in condemning sanatoria. I am fortunately able to keep in touch with a large proportion of my patients after they leave, and I am thankful to say that the great majority of them express their gratitude and appreciation of the treatment they received.

It is certainly the case at the Benenden Sanatorium, where the patients are all members of the friendly societies and other affiliated bodies to whom the beds are let, that they have every facility of complaining to their society, but I must say that in my experience such societies do not take a biased or prejudiced view of these complaints, or rush to hasty conclusions in consequence of them.

The most striking feature in connection with pulmonary tuberculosis is that we are able to do as much as we can to benefit the condition. When one thinks of the difficulties experienced in treating even small tuberculous lesions of the skin or other superficial structures, and contrasts such lesions with the comparatively enormous amount of diseased tissue in an average case of phthisis, one can be astonished and thankful that the lungs possess such marvellous recuperative powers, and that in such a large proportion of cases it is possible to restore the patient to health.

